# A New Biphasic Dicalcium Silicate Bone Cement Implant

**DOI:** 10.3390/ma10070758

**Published:** 2017-07-06

**Authors:** Fausto Zuleta, Angel Murciano, Sergio A. Gehrke, José E. Maté-Sánchez de Val, José L. Calvo-Guirado, Piedad N. De Aza

**Affiliations:** 1 Escuela de Arquitectura y Diseño, Universidad Pontificia Bolivariana, Circular 1 N° 70-01, Bloque 10 Of 306, Medellín-Antioquia 050031, Colombia; fausto.zuleta@upb.edu.co; 2 Departamento de Materiales, Óptica y Tecnologia Electrónica, Universidad Miguel Hernández, Avda. Universidad s/n, 03202-Elche, Alicante, Spain; angel@dite.umh.es; 3 Biotecnos Research Center, Rua Dr. Bonazo n° 57, Santa Maria (RS) 97015-001, Brazil; sergio.gehrke@hotmail.com; 4 Cátedra Internacional de Investigación en Odontología, Universidad Católica San Antonio de Murcia, Avda. Jerónimos, 135, 30107 Guadalupe, Murcia, Spain; jemate@ucam.edu (J.E.M.-S.d.V.); jlcalvo@ucam.edu (J.L.C.-G.); 5 Instituto de Bioingenieria, Universidad Miguel Hernandez, Avda. Ferrocarril s/n, 03202-Elche, Alicante, Spain

**Keywords:** implant surface, bone cement, bioactivity, biocompatibility, dicalcium silicate

## Abstract

This study aimed to investigate the processing parameters and biocompatibility of a novel biphasic dicalcium silicate (C_2_S) cement. Biphasic α´_L_ + β-C_2_S_ss_ was synthesized by solid-state processing, and was used as a raw material to prepare the cement. In vitro bioactivity and biocompatibility studies were assessed by soaking the cement samples in simulated body fluid (SBF) and human adipose stem cell cultures. Two critical-sized defects of 6 mm Ø were created in 15 NZ tibias. A porous cement made of the high temperature forms of C_2_S, with a low phosphorous substitution level, was produced. An apatite-like layer covered the cement’s surface after soaking in SBF. The cell attachment test showed that α´_L_ + β-C_2_S_ss_ supported cells sticking and spreading after 24 h of culture. The cement paste (55.86 ± 0.23) obtained higher bone-to-implant contact (BIC) percentage values (better quality, closer contact) in the histomorphometric analysis, and defect closure was significant compared to the control group (plastic). The residual material volume of the porous cement was 35.42 ± 2.08% of the initial value. The highest BIC and bone formation percentages were obtained on day 60. These results suggest that the cement paste is advantageous for initial bone regeneration.

## 1. Introduction

Injectability, in situ self-setting, and biocompatibility are characteristics of bone cements, which make them encouraging materials for a wide range of clinical applications in traumatology and maxillo-facial surgery.

The five main phases of Portland cement are: tricalcium silicate (Ca_3_SiO_5_), dicalcium silicate (Ca_2_SiO_4_), tricalcium aluminate (Ca_3_Al_2_O_6_), a tetracalcium alumino ferrite (Ca_4_Al_2_Fe_2_O_10_), and a sulfate phase (CaSO_4_·2H_2_O) [1]. A mixture of a refined Portland cement and bismuth oxide with a trace of SiO_2_, MgO, CaO, Na_2_SO_4_, and K_2_SO_4_ are the main components of mineral trioxide aggregate (MTA) materials [2,3]. The longer working times of the MTA material compared with those of Portland cement are because MTA contains approximately half the gypsum content of Portland cement, as well as smaller amounts of aluminum species.

MTA materials are presently used for many clinical applications in dentistry, including pulp capping, root-perforations repair, root-end filling, and apicogenesis [4,5,6,7]. The long setting time of approximately 2 h is the main disadvantage of MTA as a dental material [8]. For this purpose, bone cement should ideally have a relatively short setting time to avoid being washed away by saliva and to reduce the possibility of unset material irritating oral tissues.

It has been well-recognized that several ceramic materials which contain CaO-SiO_2_ have a high bioactive potential and are able to bond tightly to living bone [9,10,11]. Specifically, monophasic calcium silicate and β-dicalcium silicate are in vitro bioactive [12,13,14] and in vivo biocompatible [11,13,15]. Among other bioactive components, the incorporation of phosphorous into dicalcium silicate has been attempted to achieve the material’s biological anchorage, which would be promoted by the growth of bone into the pores produced by the resorption of material particles [16,17,18,19].

The isopletal section Ca_2_SiO_4_-Ca_3_(PO_4_)_2_ of the SiO_2_-CaO-P_2_O_5_ phase diagram [20,21] presents a region between 5 and 11 wt % of tricalcium phosphate, where the high temperature forms of β-dicalcium silicate and α´_L_-dicalcium silicate are stable at room temperature. Thus, the objective of this study was to investigate the processing parameter and the in vitro and in vivo biocompatibility of a novel biphasic dicalcium silicate cement.

## 2. Results

**Figure 1:**
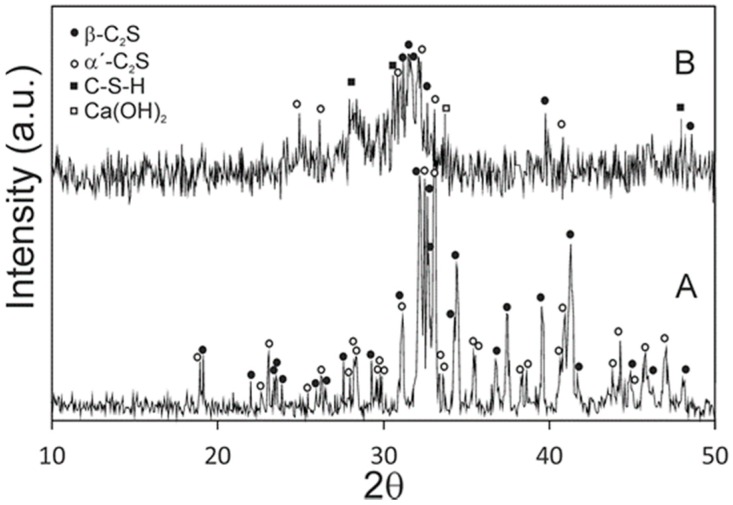
XRD patterns of (**A**) synthesized raw powder materials and (**B**) cement paste after a 24-h setting at 37 ± 2 °C and 100% relative humidity.

Figure 1 shows the XRD patterns of the synthesized powders and the cement paste after 24 h of setting at 37 ± 2 °C and 100% relative humidity. Before setting, it was obvious that the high temperature form α´_L_ and β dicalcium silicate peaks appeared, with no other detectable phases. A slight shift in relation to the corresponding JCPDS cards is observed in the XRD pattern, because the phases contain a small substitution of phosphorous-forming solid solution. After a 24-h setting, the new peaks that corresponded to calcium silicate hydrate (C–S–H) and Ca(OH)_2_ were identified from the hydration reaction of α´_L_ + β-C_2_S_ss_.

**Figure 2:**
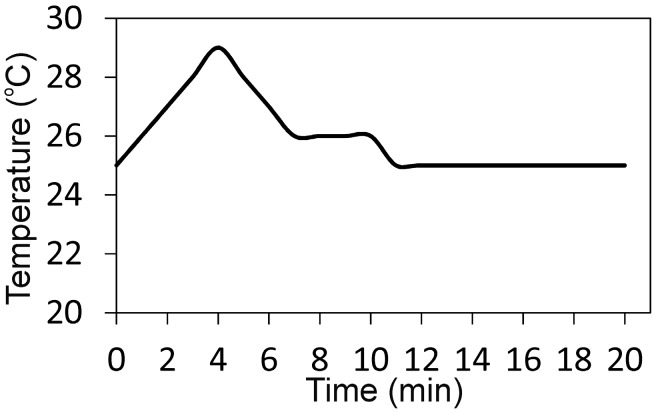
Setting temperature of the α´_L_ + β-C_2_S_ss_ paste.

The α´_L_ + β-C_2_S_ss_ paste showed short setting times (Initial (I): 35 min, Final (F): 75 min) compared with other calcium silicate cements like MTA, calcium–phosphate–silicate, and γ-dicalcium silicate [22,23,24], but were longer than those reported (I: 3 min, F: 8 min) for the calcium phosphate cements [25,26,27]. The α´_L_ + β-C_2_S_ss_ setting temperature was a slightly exhothermic process (Figure 2), but the setting temperature did not overcome the body temperature of 35.6 °C.

**Figure 3:**
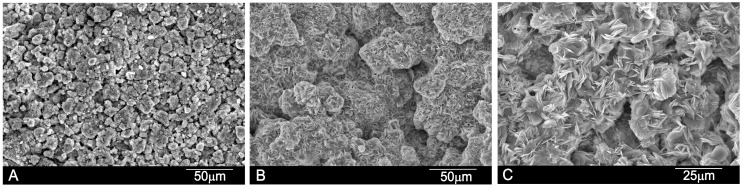
SEM images of the α´_L_ + β-C_2_S_ss_ paste with an liquid to powder ratio (L/P) ratio of 0.4 after setting for (**A**) 24 h; (**B**) 7 days and (**C**) 21 days.

Figure 3 shows the SEM images of the paste’s surface after different setting times. The SEM images reveal well-formed pores, confirmed by the shapes of the entrapped crystals. At high magnification, rod and plate-shaped crystalline C–S–H can be clearly seen in the specimens. The average pore size of the cement paste was between 25–100 μm, detected by mercury porosimetry and also microporosity, as seen in Figure 3.

**Figure 4:**
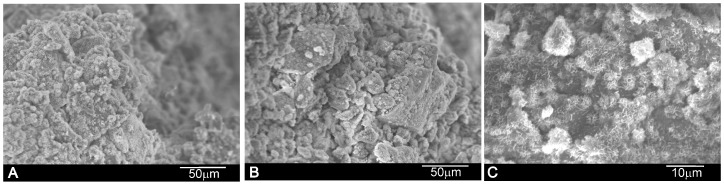
Fracture surface SEM images of the α´_L_ + β-C_2_S_ss_ paste with an L/P ratio of 0.4 after setting for (**A**) 24 h; (**B**) 7 days and (**C**) 21 days.

The internal microstructure of the paste on its fracture surfaces was studied by SEM (Figure 4). α´_L_ + β-C_2_S_ss_ hydration was favored with time, and C–S–H formed as the main product. After setting, the paste microstructure was constituted mainly of needle-like crystals, which were observed as plate-like structures randomly oriented in all directions with time. The compressive strength of the cement samples after the 21-day setting (19 ± 2 MPa) was significantly superior than that of the cement paste after the 1-day setting (10 ± 2 MPa).

**Figure 5:**
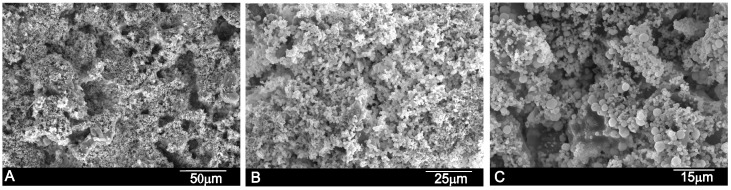
Surface SEM images of the α´_L_ + β-C_2_S_ss_ paste with an L/P ratio of 0.4 after soaking in simulated body fluid (SBF) for (**A**) 24 h; (**B**) 7 days and (**C**) 21 days.

The surface SEM images for the paste samples after immersion in simulated body fluid (SBF) are shown in Figure 5. Soaking in SBF led to distinctive surface remodeling in all of the cases. After the 24-h setting (Figure 5A), the surface of the paste exhibited the typical granular porous microstructure with the characteristic apatite morphology to those formed in the bioactive materials [12,17,28]. After prolonged soaking for up to 21 days (Figure 5B,C), clusters of agglomerated bone-like apatite particles increased and the cement’s surface structure became more compact. The EDS spectrum (data not shown) confirmed that the α´_L_ + β-C_2_S_ss_ paste surface was covered by a layer rich in calcium (Ca) and phosphorous (P). A silicon (Si) sign was also detected as a result of C–S–H formation.

**Figure 6:**
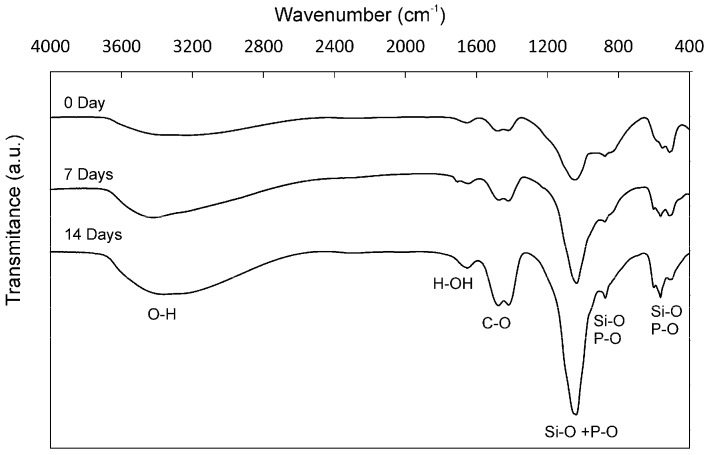
The FTIR spectrum of the α´_L_ + β-C_2_S_ss_ cement paste after 7 days and 14 days in SBF in the spectral region 400–4000 cm ^−1^. For comparison purposes, we included the FTIR spectra of the α´_L_ + β-C_2_S_ss_ cement paste after setting for 24 h.

The FTIR spectrum (Figure 6) of the α´_L_ + β-C_2_S_ss_ cement paste showed a broad peak at 3600–3200 cm^−1^ and a band at 1640 cm^−1^ before and after soaking in SBF, typical of the O–H and the H–OH stretching vibration, respectively [29], and can be assigned to the absorbed water from C–S–H. Furthermore, the C–O stretching of the CO_3_^2−^ groups at 1415 cm^−1^ and the bands between 400 and 1200 cm^−1^ were observed, which are characteristic of the vibrational modes of the Si–O–Si groups [30]. Bands between 700 and 800 cm^−1^ were observed close to the 460 cm^−1^ Si–O–Si vibration, which were identified as a flexion type vibration of the Si–O–Si group. Finally, the wide band between 1000 and 1200 cm^−1^ was associated with the asymmetrical stretching Si–O–Si ν_3_ mode. The P–O stretching vibration (ν_3_) of the PO_4_ unit occurred within the 1030–1090 cm^−1^ range. As this is overlaid with the strong vibration of the Si–O bond (the ν_3_ mode in the SiO_4_ unit), it is difficult to distinguish the P–O vibration from the Si–O vibration [31,32].

To confirm the presence of phosphorous in the cement paste before soaking in SBF, an EDS analysis was carried out. Table 1 shows the composition of the cement paste and also the composition of the raw γ-C_2_S. This finding is in good agreement with the XRD (Figure 1) results, where only the (α´_L_ + β)-C_2_S_ss_ phases are detected due the phosphorous occupying the silicon sites.

**Figure 7:**
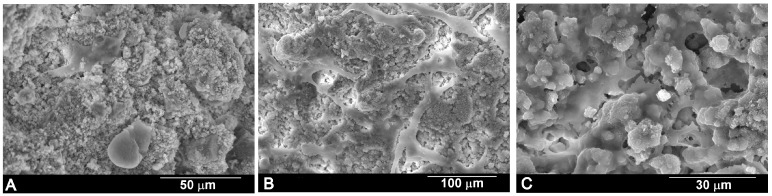
Morphological features of the human adipose stem cells (hASC) cultured on the α´_L_ + β-C_2_S_ss_ cement paste for (**A**) 1 day; (**B**) 3 days and (**C**) 10 days.

The SEM images (Figure 7) show the morphological aspect of the human adipose stem cells (hASC) cultured on α´_L_ + β-C_2_S_ss_ cement paste several times. The cells were observed to stick to the cement paste surfaces. After 1 day (Figure 7A), the majority of cells displayed a round morphology, with some cells sticking either independently or in small groups. After 3 days (Figure 7B), the density of cells increased and spread on the cement paste surface. The SEM micrographs at a higher magnification (Figure 7C) show the typical cell morphology with a spread polygonal form to form close contact with the α´_L_ + β-C_2_S_ss_ cement surface, which implies that the surface cement is a good substrate for cell attachment and growth.

**Figure 8:**
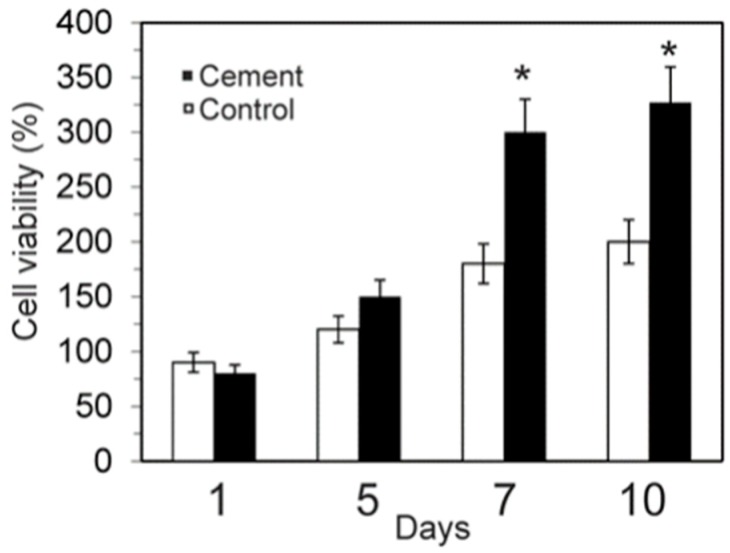
Cell viability of hASC on the α´_L_ + β-C_2_S_ss_ cement paste after culturing for 1, 5, 7, and 10 days (* significant differences *p* < 0.05).

The MTT assay (Figure 8) revealed that the dissolution extracts of the α´_L_ + β-C_2_S_ss_ cement samples were not cytotoxic against hASC. The α´_L_ + β-C_2_S_ss_ cement allowed cell growth just as well as the negative control did, and the relative cell viability of its extracts overcame 80% of that of the control for 7 and 10 days.

**Figure 9:**
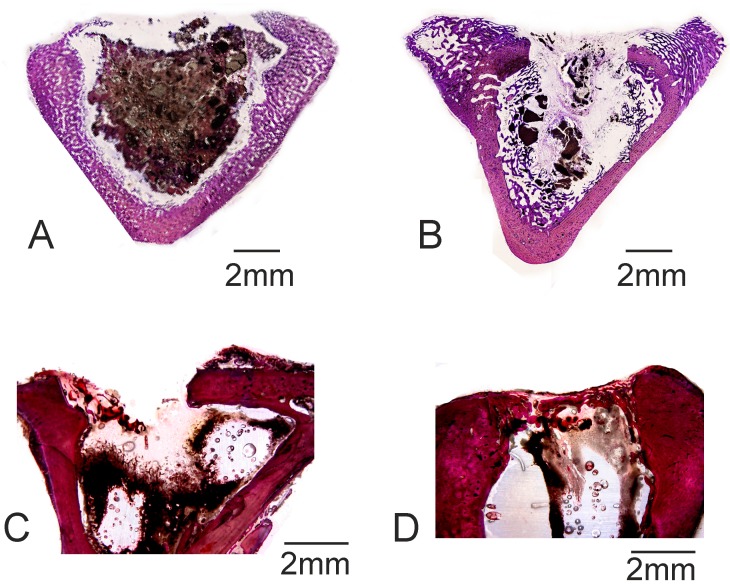
Histologic results of (**A**,**B**) the α´_L_ + β-C_2_S_ss_ cement samples and (**C**,**D**) the control group after (**A**,**C**) 30 days and (**B**,**D**) 60 days of implantation.

Figure 9 shows the histological results of the cement paste and the control defect implanted at 30 days and 60 days. The porous cement paste (Figure 9A,B) showed that resorption and bone growth, and cortex closure, were minimal compared with the other cement compositions [18,33]. Porosity was found not to be homogeneously disseminated in the porous cement paste due to the preparation and injection process in the operation theater, which should be optimized. No bone healing was observed in the control defect given the critical size (6 mm Ø), although bone remodeling and new bone formation were noted on the border of the defect (Figure 9C,D). Bone tissue remodeling was observed in the walls of the control defect on day 60, which is associated with active osteoclastic activity. Osteoid tissue was found on day 60 to always be near the walls of the defect, but almost no bone growth took place toward the center of the defect.

The histomorphometric quantification results are shown in Table 2. Analyses were run to determine the bone-to-implant contact (BIC) values for the cement paste, with high BIC values for all the analyzed times (and a closer contact). The new bone ingrowth, defect closure, and residual biomaterial were analyzed and recorded, with high values for the implant cement paste samples.

**Figure 10:**
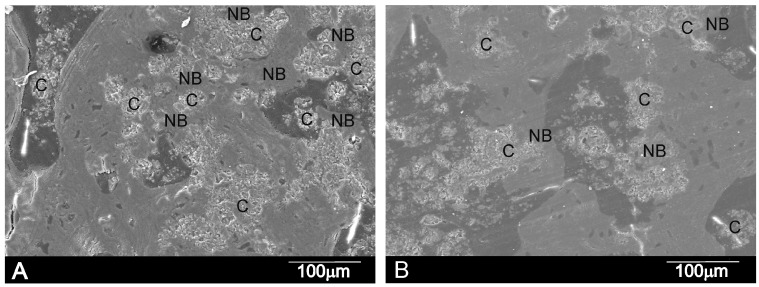
SEM micrographs of the α´_L_ + β-C_2_S_ss_ cement paste after (**A**) 30 days and (**B**) 60 days of implantation showing the cement material (**C**), interface and new bone (NB). Images show cement degradation and lack of peripheral gaps surrounding cement particles.

**Figure 11:**
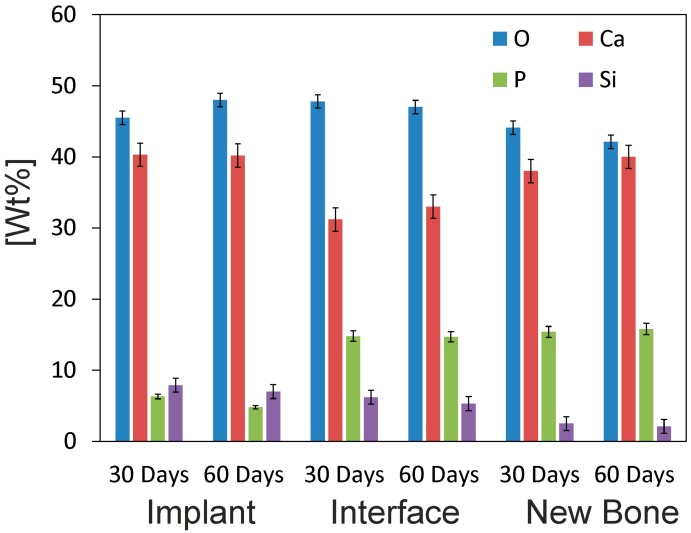
The EDS analysis of the α´_L_ + β-C_2_S_ss_ cement paste-bone interface after 30 and 60 days of implantation. The Wilcoxon test was run to compare means by assuming a significance level of 95% (*p* < 0.05).

The SEM micrographs showed that the cement paste had completely integrated into the implant site (Figure 10). The details in each image reveal that the cement paste showed both a degradation process and cellular arrangement over the implant. The EDS microanalysis results (Figure 11) indicated the partial dissolution of the cement implant due to Si lowering (from 8.3 to 7.0), as well as the P ion concentration (from 6.9 to 4.8) from the implant. The newly grown bone surrounding the implant did not achieve the same degree of mineralization within a distance from the interface. The relative Ca/P ratio in the new bone area, around 80 μm away from the interface, obtained a value of 2.53. In the areas closer to the implant, the ratio consistently lowered to 2.1 at the interface.

## 3. Discussion

The applicability of self-setting biocements is largely dependent on their self-setting characteristics, such as injectability and setting times. Dicalcium silicate has a self-setting property due to the gradual hydration of SiO_4_^4−^ ions in dicalcium silicate. It has been well-recognized that when dicalcium silicate reacts with water, an amorphous nanoporous C–S–H gel is formed, and Ca(OH)_2_ crystals nucleate and grow in the existing capillary pore area in an earlier formed C–S–H gel. As time proceeds, the C–S–H gel polymerizes and hardens.

The primary binding phase in Portland cement is the calcium silicate hydrate (C–S–H) phase [1,34], and the C–S–H in our cement paste is amorphous or semicrystalline, which makes it difficult to determine its stoichiometry (Figure 1). The chemical composition of the C–S–H phase during the hydration process of dicalcium silicate has been the topic of several papers [35,36], because its poorly crystalline feature makes it difficult to characterize. In agreement with Taylor [1], there are two types of C–S–H phases according to their Ca/Si ratio. Below 1.0 is the C–S–H (I) from a tobermorite model, and above 1.0 is the C–S–H (II) from a jennite model. In our case, the chemical composition of the C–S–H phase, formed due to cement paste hydration (Figure 3), came close to the Ca_5_Si_2_O_9_·H_2_O phase, with a molar ratio of Ca/Si > 1, which matches the C–S–H (II) family.

In vitro bioactivity evaluation was an essential first step before assessing in vivo the bone cement’s performance. After soaking in SBF for a short time (e.g., 24 h), the cement samples induced the precipitation of apatite spheres (Figure 3), which indicates the cement’s high bioactivity. To confirm that the observed apatite spheres were certainly precipitated from SBF, an EDS microanalysis and FTIR (Figure 6) were performed on the immersed surface.

As far as we know, this study is the first to describe the in vivo achievement of a biphasic dicalcium silicate macroporous cement (Figure 3) prepared and implanted in situ. One of the most serious forms of damage to be caused to bone cements is fragmentation, or their inability to set once they come into contact with body fluids, which can imply an inflammatory response [37]. The cement paste used herein had to resist blood pressure and prevent pore failing, and also required good resistance to water penetration and fragmentation before the setting reaction was completed.

The study also showed the good osteoconductivity of the biphasic dicalcium silicate macroporous cement. Neither fibrous tissue development nor inflammatory response, which have been described in other calcium phosphate cement pastes in some subcutaneous or intramuscular implantations [38], and also in brushite pastes [39,40], were observed in this study.

By day 30, bone tissue was found to grow in close contact with the α´_L_+β-C_2_S_ss_ cement paste. Neovascularisation and bone growth were observed inside pores and on the cement paste margins, as evidenced by a higher new bone ingrowth value (see Table 2). Bone growth took place mainly in pores, but did not in fact occur only on the cement paste margins because it also grew in some central pores. An absence of more extensive bone tissue penetration in the central part of the cement implant by day 30 was due mainly to the reduced interconnectivity between the adjacent pores within the 50–150 μm range. Mercury porosimetry detected an average pore size of between 50–100 μm. A minimum pore size of 100 μm has been described to allow bone tissue formation inside the pores of materials [41,42], and the optimum pore size falls within the 200–400 μm range. Nevertheless, the most important factor is pore interconnectivity. Additional smaller pores have also been described as being useful for body fluid circulation [43,44]. In our case, this was guaranteed by the intrinsic microporosity of the α´_L_ + β-C_2_S_ss_ cement paste (Figure 3). The residual biomaterial value had already lowered by day 30, and more significantly by day 60 after implantation (Figure 9A,B and Table 2). Bone was formed and started to grow from the cement paste surface, which indicates osteostimulative behavior. By day 60, the α´_L_ + β-C_2_S_ss_ cement paste had almost been reabsorbed (Figure 9B).

The SEM confirmed the histological conclusions. Figure 10 demonstrates the substantial new bone colonization of the α´_L_ + β-C_2_S_ss_ cement paste through the original pores in the cement paste due to gradual structure dissolution. As a result of these advanced processes, cement paste fragments were found in many areas and all over the restructuring implant. (C) and (NB), respectively, represent the cement paste particles and ingrown bone regions in Figure 10.

Two resorption mechanisms take place in bioceramic materials: passive resorption due to the dissolution of the ceramic material, and active resorption due to osteoclast and macrophage activity [45]. Both mechanisms probably took place in the α´_L_ + β-C_2_S_ss_ cement paste. The dissolution of the cement paste could improve given the larger contact area with body fluids, which could encourage cement fragmentation. Figure 10 shows the cement single paste particles on days 30 and 60 after implantation due to the degradation of the cement surrounded by bone tissue.

## 4. Materials and Methods

### 4.1. Synthesis of the Powder and Cement Paste Preparation and Characterization

The biphasic α´_L_ + β dicalcium silicate powder was prepared from the γ-dicalcium silicate and β-tricalcium phosphate ceramic powders synthesized in our laboratory according to previously reported protocols [13].

The γ-dicalcium silicate and β-tricacium phosphate, in the 92:8 wt % ratio, were crushed and dusted in an attrition-mill with isopropilic alcohol as the liquid medium and ZrO_2_-Y_2_O_3_ balls (1 mm diameter) for a 2-h period at 500 rpm. This was then isostatically pressed into bars at 200 MPa and heated up to 1500 °C during a 5-day period, with quenching in liquid-nitrogen, milling, pressing, and reheating every 24 h. Finally, the sample was cooled from 1500 °C inside the furnace to 400 °C, and heated at 400 °C/5 days. Then, the powder was turned off and the ceramic was allowed to cool inside the furnace for 24 h. This combined heat treatment procedure was required to ensure equilibrium conditions. The heat treatment temperatures were carefully selected by bearing in mind the information provided by the 2CaOSiO_2_-7CaOP_2_O_5_2SiO_2_ sub-system [21], which exists in the binary system of Ca_3_(PO_4_)_2_-Ca_2_SiO_4_ [20]. The obtained ceramic was ground and characterized by X-ray diffraction (XRD).

To prepare the cement pastes, the α´_L_ + β dicalcium silicate powder was manually mixed with distilled and deionized water at a liquid to powder ratio (L/P) of 0.40 mL/g. The mixtures were stirred to form homogeneous pastes within 1 min by a stainless-steel spoon, transferred to Teflon molds, and cured in a 100% humidity environment at 37 ± 2 °C.

The crystalline phases present in the raw materials and set cements were identified in an AXS D8Advance XRD (Bruker, Karksruhe, Germany), and compared with the database provided by the Joint Committee on Powered Diffraction Standards (JCPDS). DTA (Model STA 409, Selb, Germany). Fourier transform infrared spectroscopy (FTIR) was performed to determine the various functional groups present in the setting cement pastes. The IR measurements were taken by an IF66a Bruker spectrophotometer (Karksruhe, Germany) within the wavenumber range from 400 to 4000 cm^−1^.

The initial and final setting times were measured with the Vicat needle according to UNE-EN 196-3:2005+A1 (Methods of testing cements. Part 3: Determination of setting times and soundness). The cement setting temperature was recorded by an AZ8895 High Temperature Infrared Meter (AZ Instrument, Taichung City, Taiwan). At least three measurements were taken for the cement pastes. The maximum temperature reached during setting was measured for at least five replicas, and the mean value was calculated.

For the compressive strength measurements, cement pastes were cast into teflon moulds to form standard test cylindrical probes (6 mm Ø and 12 mm in height). Ten probes were prepared and aged in a 100% humidity environment at 37 ± 2 °C for 1 day and 21 days, and the results were expressed as mean ± standard deviation (mean ± SD). Measurements were taken in a universal testing machine (Instron, model 1195, Norwood, MA, USA) according to ASTM D695-91, provided with a load cell of 5 kN at a loading rate of 100 N sg^−1^.

The surface and broken surfaces of the cement pastes, both before and after being tested in compression, were characterized under a scanning electron microscope (SEM-Hitachi S-3500N, Ibaraki, Japan) at an accelerating voltage of 20 kV. The cement pastes prior to observations were palladium-coated.

### 4.2. In Vitro Bioactivity

Simulated body fluid (SBF) was prepared according to a procedure described elsewhere [12,19]. Cement paste samples (discs with a 6-mm diameter and a 2-mm thickness) were placed inside polystyrene vials that contained SBF, and were maintained at 37 ± 2 °C for 24 h, 7 days, and 21 days without refreshing the soaking medium. The samples were gently rinsed with deionized water to remove SBF solutions, and were dried at room temperature. Any morphological variations in the disc surfaces were analyzed by SEM-EDS.

### 4.3. Cell Test

A culture medium of human adipose stem cells (hASC) was used to evaluate the in vitro biological response of the α´_L_ + β-C_2_S_ss_ cement paste. The cell proliferation assay was performed by the extraction method with hASC isolated from the subcutaneous adipose tissue of volunteer female donors who had undergone elective liposuction procedures. Informed consent was obtained from all of the volunteers. All of the procedures were approved by the UMH-Ethics Committee (approval ID: 2014/VSC/PEA/00056 tipo2). Samples were collected from three different patients aged 25–35 years. Details of the method and technique used to obtain, subculture, and characterize the cells prior to seeding on the cement paste can be found in previous publications [14,46].

The 3-(4,5-dimethylthiazol-2-yl) 2,5-diphenyl tetrazolium bromide (MTT) method was used to assess cell proliferation levels. This assay relies on the ability of living cells to reduce a tetrazolium salt into a soluble colored formazan product. To determine the toxicity of the leached, samples were prepared according to previously reported protocols [47]. Plastic was used as control in the present assay.

hASC were grown in the α´_L_ + β-C_2_S_ss_ cement paste in 700 cells/mm^2^. The medium was replaced every 2 days during the course of the experiment (1–10 days). The sample analysis results were obtained in triplicate from three separate experiments.

The surface morphology of the specimens was analyzed by SEM-EDS to evaluate cell growth and adhesion to the α´_L_ + β-C_2_S_ss_ cement paste surface. After incubation for 1, 3, and 10 days, samples were removed from the culture well, rinsed with PBS, and fixed with 3% glutaraldehyde in a 0.1 M cacodylate buffer for 1.5 h at 4 °C. Then they were rinsed and post-fixed in osmium tetroxide for 1 h before being dehydrated at increasing ethanol concentrations with a final dehydration in absolute alcohol. Before the cell culture studies, and in order to recognize the seeded cement paste surface, one of the sample faces was carefully impressed with an electrical marker. The specimens were dried by the critical-point method, and palladium-coated and examined by SEM-EDS according to previously reported SEM protocols [14].

### 4.4. Animal Test

The study protocol was approved by the Animal Ethics Committee of the Miguel Hernandez University, which followed Spanish Government and European Community Guidelines for animal care (authorized No. 2014/VSC/PEA/00056 tipo2). The study used 15 male New Zealand rabbits that weighed 3.5–4.5 kg. The cement paste was implanted into critical size defects in the animals’ tibiae. The total sample size was 15 rabbits with two defects in each tibia, which totaled 60 defects, divided randomly into two groups of 30: test (cement paste) and control (randomization). The surgical procedure and the sacrificed animals have been previously reported by our group [43,48].

### 4.5. Cement Implant Characterization

The cement implants, together with the surrounding tissue, were removed after 30 days and 60 days of implantation, and were fixed in 10% neutral buffered formalin and decalcified. The utilized decalcification method was Osteomoll Merck KbaA (Darmstadt, Germany) by soaking the samples for 17 days and renewing the solution every 24 h. Subsequently, all of the samples were paraffin-embedded, sectioned at a 5-μm depth, and stained using hematoxylin-eosin. The entire circumference of each section, which contained bone, implant, and connective tissue, was traced manually to create an individual region of interest (ROI).

The standardized nomenclature of the American Society of Bone and Mineral Research was used for the histomorphometric evaluations with the Image J software (developed by the National Institute of Health, Bethesda, MD, USA). Examinations were made under a Nikon Elipse 80i microscope (Teknooptik AB, Huddinge, Sweden), equipped with the Easy Image 2000 system (Teknooptik AB), which used 10× to 40× lenses for the descriptive evaluations and morphometric measurements. Images were generated with a Leica Z6 APO microscope connected to a Leica DC 500 (Barcelona, Spain) digital camera, enlarged 23×. After calibrating the system and digitalizing the images, the interactive measurements of the individual ROIs were obtained by the Leica QWin V3 image analysis software (Barcelona, Spain). The histomorphometric analysis produced one BIC measurement, measured as the percentage of the circumference and the length of the cylinder that came into contact with new bone.

The cement’s resorption rate was determined by an Image J image analysis program (National Institutes of Health, Bethesda, MD, USA) by measuring the perimeter of the cement paste after implantation and comparing it with the residual cement after 15, 30, and 60 days.

To evaluate the continuing effect of the cement implant from an ultrastructural point of view, cross-sections of the non-decalcified tissues were examined in SEM-EDS according to previously reported SEM protocols [43,48].

### 4.6. Statistical Analysis

A statistical analysis was performed using the PASW Statistics v.20.0.0 software (SPSS Inc, Armonk, NY, USA). Values were recorded as means +/− standard deviation and medians. A Wilcoxon Test was run to compare the means by assuming a significance level of 95% (*p* < 0.05). Equal means were regarded as the null hypothesis, while the existence of significant differences between means acted as an alternative hypothesis. As significant differences existed between the means, the null hypothesis was rejected.

## Figures and Tables

**Table 1:** EDS analysis of the cement paste before soaking in SBF and a raw γ-C_2_S ceramic for comparative purpose.

wt %	Ca	P	Si	O
γ-C_2_S	46.54	-	16.29	37.16
Cement paste	45.92	1.59	15.01	37.48

**Table 2:** Histomorphometric analysis to evaluate the bone-to-implant contact (BIC) for the α´_L_ + β-C_2_S_ss_ cement implant.

Days/(%)	Cement Paste Implant		Control	
	30 Days	60 Days	30 Days	60 Days
	Mean ± SD	Mean ± SD	Mean ± SD	Mean ± SD
BIC	48.67 ± 0.83 *	55.86 ± 0.23 *	0.00 ± 0.00	0.00 ± 0.00
New bone ingrowth	54.96 ± 2.46 *	65.53 ± 2.86 *	17.26 ± 2.73	26.17 ± 1.75
Defect closure	42.46 ± 2.48 *	58.94 ± 2.48 *	28.46 ± 2.07	30.86 ± 1.86
Residual biomaterial	45.04 ± 1.67	35.42 ± 2.08	0.00 ± 0.00	0.00 ± 0.00
Resorption rate	45.21 ± 2.64	47.31 ± 2.62	0.00 ± 0.00	0.00 ± 0.00

A Wilcoxon test was run to compare means by assuming a significance level of 95% (*p* < 0.05). Abbreviation: SD, standard deviation (* significant differences *p* < 0.05).
